# Ion-Specific Effects of Alkaline Earth Metal Ion Binding to an Anionic Carboxylate Monolayer

**DOI:** 10.1021/acs.langmuir.5c04163

**Published:** 2025-12-24

**Authors:** Lacey LaBee, Kierra Parker, Audra Dempsey, Minh Tran, Gabby Delpleash, Ann Obiesie, Desirè Johnson, R. Sydney Williams, Makenzie Provorse Long

**Affiliations:** † Department of Chemistry and Biochemistry, 2673 University of Central Arkansas, Conway, Arkansas 72035, United States; ‡ Department of Chemistry and Biochemistry, 6216 Creighton University, Omaha, Nebraska 68178, United States

## Abstract

The ion-specific effects of alkaline earth metal ions binding to anionic carboxylate-terminated monolayers influence environmental chemistry, industrial processes, and nanotechnology. Experimental results obtained using interfacial techniques are often interpreted by using data from dilute aqueous solutions of carboxylate anions. However, atomic force microscopy (AFM) adhesion forces reported for alkaline earth metal ions binding at the aqueous interface of an anionic carboxylate-terminated monolayer contradict data reported for dilute solutions of aqueous carboxylate anions. To reconcile this data and provide molecular insight into the ion-specific trends observed in the AFM data, classical atomistic molecular dynamics (MD) simulations are used to model Mg^2+^, Ca^2+^, Sr^2+^, and Ba^2+^ ions at the aqueous interface of a deprotonated 11-mercaptoundecanoic acid (MUA) monolayer. We compare site-specific and collective ion binding approaches for calculating the strength of ion binding at the carboxylate interface. This comparison reveals that site-specific approaches (i.e., simulated adhesion force curves and potential of mean force binding free energies) qualitatively agree with dilute aqueous solution data, whereas a collective ion approach (i.e., binding free energies from the Langmuir isotherm) is necessary to reproduce experimental AFM results. Analysis of the validated MD simulations reveals that Ca^2+^ and Ba^2+^ ions more efficiently occupy the carboxylate interface, which leads to a larger portion of ions binding to the monolayer through direct contacts, forming contact ion pairs with multiple MUA ligands. These results rationalize the ion-specific results reported by AFM studies and may be used to inform future studies of interfacial processes involving alkaline earth metal ions and carboxylate-functionalized structures.

## Graphical abstract

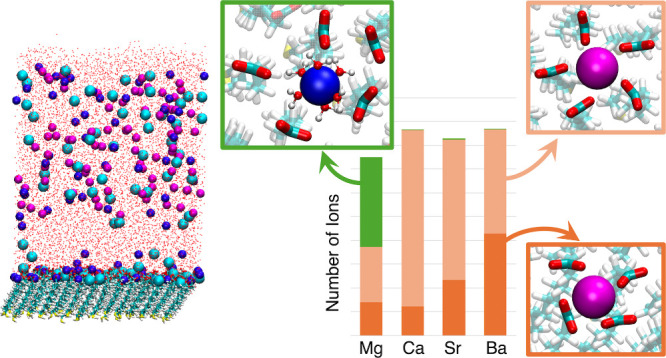

## Introduction

Alkaline earth metal ions interact with interfacial carboxylate groups in many applications relevant to environmental chemistry, industrial processing, and nanotechnology. Mg^2+^ and Ca^2+^ ions selectively enrich sea spray aerosols, which influence Earth’s air quality and climate patterns.^1–3^ This enrichment may occur due to alkaline earth metal ion complexation with fatty acids within the sea surface microlayer.^4,5^ Mg^2+^ and Ca^2+^ also promote the adsorption of anionic soft matter, such as proteins and alginate, to deprotonated fatty acid monolayers.^6–8^ Crude oil processing is hampered by organic deposits formed by the self-assembly of tetrameric acid vesicles induced by Ca^2+^ ion complexation.^9^ The ability of alkaline earth metal ions to induce self-assembly and adsorption of nanoscale materials is harnessed in nanotechnology. For example, Mg^2+^, Ca^2+^, and Ba^2+^ exhibit ion-specific effects on the self-assembly of protein nanostructured lattices.^10^ Sr^2+^ and Ba^2+^ ions induce reversible formation of micelles from carboxylate-functionalized polymers.^11^ Divalent metal ions also induce adsorption of DNA to charged monolayers with ion-specific effects.^12–16^

Many of these applications involve the interaction of alkaline earth metal ions with a carboxylate-terminated monolayer, such as deprotonated palmitic acid or 11-mercaptoundecanoic acid (MUA). Vazquez de Vasquez et al. studied the interfacial process of Mg^2+^ and Ca^2+^ binding to a palmitic acid monolayer using infrared reflection–absorption spectroscopy (IRRAS) and Raman spectroscopy to monitor the carboxylate vibrational modes.^17^ This interfacial data was interpreted using Raman spectroscopy and density functional theory (DFT) molecular dynamics (MD) simulations of a dilute acetate aqueous solution. Their work suggests that Ca^2+^ binds to the carboxylate-terminated monolayer directly through contact ion pairs and indirectly through solvent-shared ion pairs, whereas Mg^2+^ ions remain fully hydrated and primarily bind to the monolayer indirectly through solvent-shared ion pairs.

Dilute solutions of small model compounds are often used to interpret the interfacial data. Denton et al. provides experimental and computational evidence that the vibrational modes of small carboxylate ions can be extrapolated to those of the aqueous interface of a carboxylate-terminated monolayer as the size of the hydration shell increases.^18^ This approach was applied to Ca^2+^ and Mg^2+^ ions directly bound to small carboxylate ions through bidentate contacts.^18,19^ Mendes de Oliveira et al. used Raman spectroscopy to measure binding constants of Mg^2+^ and Ca^2+^ directly bound to aqueous acetate in a one-to-one ratio.^20^ Sigel and McCormick reported one-to-one binding constants for Mg^2+^, Ca^2+^, Sr^2+^, and Ba^2+^ to small carboxylate ions using potentiometric titrations.^21^ In agreement with DFT calculations,^22,23^ the binding constants reported by Mendes de Oliveira et al.^20^ and Sigel and McCormick^21^ indicate that the strength of the Mg^2+^-carboxylate interaction is stronger than the Ca^2+^-carboxylate interaction when measured for a dilute acetate aqueous solution.

Direct measurement of the strength of the ion-carboxylate interaction at an aqueous interface was reported by Rios-Carvajal et al. using atomic force microscopy (AFM).^24,25^ In this work, a bare gold substrate and the AFM tip were functionalized with an alkanethiol carboxylate-terminated self-assembled monolayer (i.e., MUA). The AFM adhesion force was reported for a series of aqueous alkaline earth metal ion salt solutions. The reported AFM adhesion forces reveal that the strength of the alkaline earth metal ion interaction with a carboxylate-terminated monolayer increases as Mg^2+^ < Sr^2+^ < Ca^2+^ < Ba^2+^.^24^ This result is consistent with the interfacial infrared spectroscopy experiment of Vazquez de Vasquez et al. in that Ca^2+^ ions bind more strongly through direct contact ion pairs than the solvent-shared ion pairs formed by Mg^2+^ ions.^17^ However, this trend is opposite those reported by experimental and computational studies of dilute aqueous solutions of small carboxylate ions, such as acetate.^20–23^

Currently, there is a discrepancy between the interfacial data for alkaline earth metal ion binding at the aqueous interface of anionic carboxylate-terminated monolayers and the dilute aqueous solution data often used to interpret the interfacial data. We aim to reconcile these seemingly disparate results using classical atomistic MD simulations. This method provides atomic-resolution of ion binding motifs, including bidentate and monodentate direct binding (i.e., contact ion pairs) and indirect binding (i.e., solvated-shared ion pairs). Previous MD simulations have used DFT, a quantum mechanical method, to model ion-carboxylate binding, but this approach is restricted to small molecular systems due to computational cost.^18,23^ Classical MD simulations are computationally efficient enough to model aqueous interfaces of monolayers and bilayers.^26–28^ There are many examples in the literature that use classical MD simulations to model monovalent ion adsorption at the aqueous interface of carboxylate-terminated monolayers (e.g., Szefczyk et al.,^27^ Schwierz et al.,^29^ and Li et al.^30^), but few studies model divalent metal ion adsorption (e.g., Duffy and Harding^31^). Parameterization efforts have improved the ability of classical force fields to accurately model divalent metal ion binding to charged species,^20,32–34^ but these parameters have not been tested for accurate simulation of divalent metal ion binding at the aqueous interface of a carboxylate-terminated monolayer. Our work addresses this gap in the literature by employing a variety of alkaline earth metal ion force fields parametrized against local divalent metal ion properties and testing their ability to reproduce quantitative data from interfacial AFM experiments.

An alternative approach to modeling ion adsorption at the aqueous interface of a charged monolayer is to use a theoretical model, such as Poisson–Boltzmann theory, with or without modifications to account for ion and solvent effects.^29,35^ The review article by Judd et al. describes commonly used theoretical models and discusses their strengths and limitations within the context of aqueous interfaces of soft matter, such as Langmuir monolayers.^36^ Schwierz et al. used atomistic simulation data as input for a modified Poisson–Boltzmann theoretical model to investigate monovalent ion adsorption to a carboxylic acid terminated monolayer.^29^ Theoretical models and experiments demonstrate that excess multivalent cations adsorbed to an anionic monolayer can invert the electrostatic potential of the interface from negative to positive values, called charge inversion or overcharging.^37–39^ Both ion correlation and site-specific ion binding mechanisms are used to explain the origin of multivalent ion adsorption, including ion specific effects.^39–44^ These results are dependent on system conditions, such as the multivalent ion concentration, surface protonation, cation identity, and monolayer functionalization. Theoretical models that account for ion correlation and excluded volume or steric effects can reproduce concentration- and pH-dependent interfacial properties.^35,37,38^ However, ion specific effects, especially for divalent metal ions, remain a challenge for many theoretical models.

Here, we employ atomistic simulations to provide physical insight into the mechanism of divalent metal ion adsorption at the aqueous interface of a carboxylate-terminated monolayer. A deprotonated MUA monolayer solvated by aqueous solutions of Mg^2+^, Ca^2+^, Sr^2+^, or Ba^2+^ ions is modeled using classical MD simulations. Simulated results are computed using various alkaline earth metal ion force fields that were parametrized to reproduce different local ion properties, such as the ion hydration free energy and the ion–water oxygen distance.^20,32,33^ By comparing these simulated results to experimental AFM data, this work explores the validity of employing these classical force fields to model divalent ion adsorption at the aqueous interface of a carboxylate-terminated monolayer. The interface of a functionalized AFM tip and a functionalized surface is more complex than this simple ion-monolayer system.^24,45^ However, using an ion-monolayer system in our MD simulations allows us to investigate the accuracy of divalent metal ion force fields for modeling ion-carboxylate interactions at the monolayer interface without the complexity of accurately modeling the AFM tip. An additional benefit of using the simple ion-monolayer system is that the factors that influence the adsorption behavior of divalent metal ions at the monolayer are disentangled from the more complex ion-bridging or correlated ion mechanisms that may be at play when the divalent metal ions facilitate adsorption of a nanoscale structure, such as an AFM tip. Comparing results from this work to future simulations of more complex systems, such as a functionalized AFM tip interacting with a functionalized surface, will provide additional physical insight into the adsorption behavior of divalent metal ions at aqueous interfaces of anionic monolayers.

In this work, the mechanism of alkaline earth metal ion binding to a carboxylate-terminated monolayer is investigated by comparing site-specific and collective ion binding approaches to quantifying the ion binding strength. These approaches differ in the physical properties used to quantify the ion binding strength. Site-specific approaches sample local configurations of ions, whereas the collective ion binding approach focuses on the equilibrium distribution of the ions. Comparing the ion binding strength computed using these approaches to the AFM results provides physical insight into the factors that influence the strength of ion-carboxylate binding at an aqueous interface. Analysis of the ion-monolayer simulations reveals the origin of ion-specific effects of alkaline earth metal ion binding at the aqueous interface of a carboxylate-terminated monolayer. This analysis provides physical insight into the ion-specific effects reported by previous AFM and interfacial vibrational spectroscopy experiments.

## Materials and Methods

### Simulation Details

A deprotonated 11-mercaptoundecanoic acid (MUA) self-assembled monolayer was simulated using 154 MUA ligands in a 14 × 11 array (Figure 1). Initial coordinates for the MUA ligand were downloaded from the Web site of Latour.^46^ The sulfur atoms of the MUA ligands were restrained to the experimentally determined (√3×√3)­R30° lattice structure^47^ using harmonic restraints with a force constant of 50,000 kJ/mol·nm^2^ in each direction. The MUA ligands were deprotonated and modeled using the CHARMM Generalized Force Field (CGenFF).^48^ The MUA monolayer was solvated with a 60.6 Å × 52.0 Å × 70.0 Å water box containing ∼7,000 TIP3P water molecules,^49^ 100 divalent metal cations (Mg^2+^, Ca^2+^, Sr^2+^, or Ba^2+^), 54 Na^+^ ions, and 100 or 60 Cl^–^ ions (Figure 1c). The monovalent ions were modeled using the ion parameters distributed in the November 2018 version of the CHARMM36 force field for GROMACS (Table S1). The solvated monolayer was simulated using periodic boundary conditions in all directions. A large slab of vacuum at least three times the height of the solvated monolayer was added in the *z*-direction (i.e., the vector normal to the MUA monolayer surface) to separate the periodic images in this direction.

**Figure 1:**
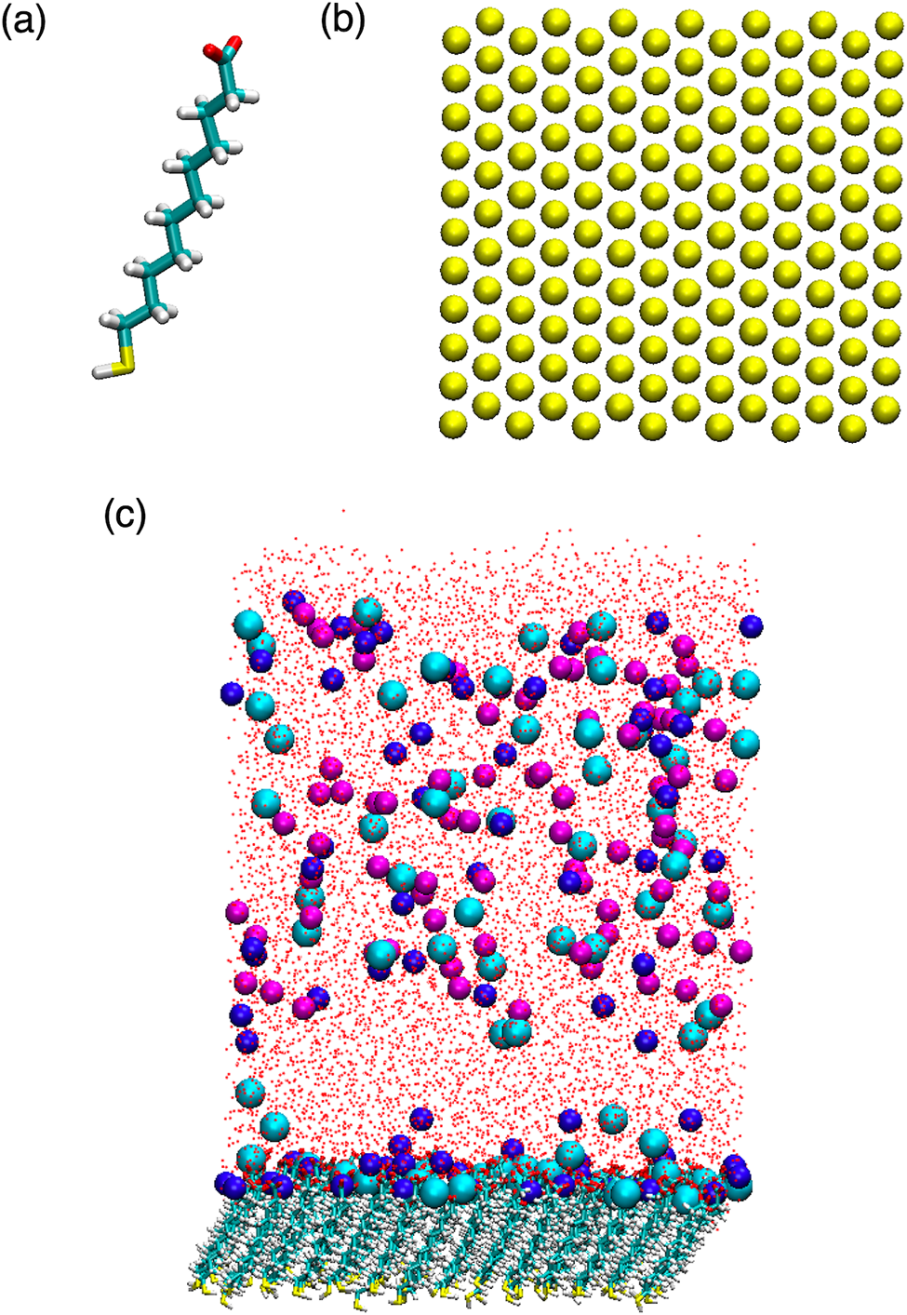
(a) Structure of a MUA ligand shown in the licorice representation where S, O, C, and H are yellow, red, cyan, and white, respectively. (b) Two-dimensional array of sulfur atoms in the MUA monolayer, where S atoms are shown as yellow spheres. (c) Side view of the solvated MUA monolayer, where Mg^2+^, Na^+^, and Cl^–^ ions are shown as blue, cyan, and magenta spheres, respectively, and water oxygen atoms are shown as red dots.

The solvated monolayer was relaxed using steepest descent to converge the atomic forces to less than 1000 kJ/mol·nm. Subsequent temperature coupling using the velocity rescaling thermostat^50^ heated the system from 100 to 300 K. Semi-isotropic pressure coupling was performed with the Parrinello–Rahman barostat^51^ to equilibrate the pressure in the *z*-direction to approximately 1 bar, while maintaining constant surface area in the *x*- and *y*- directions and constant temperature at 300 K using the velocity rescaling thermostat. Production simulations were performed using the velocity rescaling thermostat and the box vectors from the last step of the semi-isotropic pressure coupling simulation to simulate a constant number of particles, volume, and temperature (NVT) ensemble at 300 K. The alkaline earth metal ion concentration was approximately 0.65 M in each production simulation. The equilibration and production simulations used a 2 fs time step with the SETTLE and P-LINCS algorithms^52,53^ to restrain hydrogen-containing covalent bonds in water and the MUA monolayer, respectively. Long-range electrostatic interactions were computed using particle mesh Ewald summation^54^ with a 12 Å cutoff distance. Long-range van der Waals interactions were computed by using a force-switch transition from 10 to 12 Å.

Equilibration and a 200 ns production simulation was performed for each divalent metal cation (Mg^2+^, Ca^2+^, Sr^2+^, or Ba^2+^). The number of divalent metal ions bound to the MUA monolayer converged near 100 ns of simulation time (Figure S1). The last 100 ns of each production simulation was used for analysis. The average number of ions bound for the last 100 ns of each simulation has a standard deviation of about one divalent metal ion (Table S2). This simulation procedure was validated by reproducing Ca^2+^-carboxylate distances from *ab initio* simulations^55^ and the MUA tilt angle for Na^+^ simulations in which no divalent metal ions were included^27^ (Table S3). All classical atomistic MD simulations were performed using GROMACS 2022 or 2024.^56^ Analysis of the production simulations was performed using the GROMACS analysis tools.

### Divalent Metal Ion Force Fields

Each divalent metal ion is modeled using a nonbonded potential energy function (*U*_*ij*_) that uses a 12-6 Lennard-Jones potential to model van der Waals interactions and a Coulomb potential to model electrostatic interactions,

1 $$U_{ij}\left(r_{ij}\right)=\varepsilon_{ij}\left[{\left(\frac{R_{\min,ij}}{r_{ij}}\right)}^{12}-2{\left(\frac{R_{\min,ij}}{r_{ij}}\right)}^{6}\right]+\frac{e^{2}Q_{i}Q_{j}}{r_{ij}}$$

where *r*_*ij*_ is the distance between atoms *i* and *j*, *ε*_*ij*_ is the Lennard-Jones potential well-depth, *R*_min,*ij*_ is the distance between atoms at the minimum of the Lennard-Jones potential, *e* is elementary charge, and Q_*i*_ and Q_*j*_ are the point charges of *i* and *j*. Note that *R*_min,*ij*_ may be expressed in terms of $\sigma_{ij}=R_{\min,ij}/2^{1/6}$ . The attraction between divalent metal ions and charged species is likely overestimated using standard 12-6 Lennard-Jones and Coulomb potentials.^20,34^ To improve force field accuracy, an electronic continuum correction (ECC) may be applied that scales the Coulomb potential by $f=1/\sqrt{\varepsilon_{\mathrm{el}}}$ , where *ε*_el_ is the electronic dielectric constant of the solvent.^57^ Alternatively, a 12-6-4 Lennard-Jones potential may be used that includes a third term in the Lennard-Jones potential to model charge-induced dipole interactions, which effectively scales the van der Waals interactions rather than the electrostatic interactions of the metal ion. Li and Merz demonstrated that their 12-6-4 Lennard-Jones potential reproduces hydration free energies of metal ions to within 1%.^34^

Here, we choose to use the well-established 12–6 Lennard-Jones potential and test different parametrizations for alkaline earth metal ions with and without ECC charge scaling (Table 1). Many 12-6 Lennard-Jones force fields have been proposed for Mg^2+^ and Ca^2+^,^32–34,58–62^ but only a few include Sr^2+^ and Ba^2+^.^32–34^ The Li et al. force field referenced in Table 1 is the compromise (CM) set of parameters that aim to reproduce experimental hydration free energies and ion coordination numbers for the TIP3P water model.^33^ Similar parameters optimized for the SPC/E water model^33^ were previously evaluated against experimental binding free energies of ion-acetate binding in aqueous solution for Mg^2+^ and Ca^2+^.^20^ The Mamatkulov and Schwierz force field referenced in Table 1 was parametrized to reproduce not only experimental hydration free energies, but also the activity coefficient and water residence time of divalent metal ions.^32^ This additional experimental reference data may provide more accurate divalent metal ion force field parameters; however, this force field has not been tested using ECC charge scaling nor for reproducing ion-acetate binding free energies. The Mendes de Oliveira et al. force field referenced in Table 1 is limited to Mg^2+^ and Ca^2+^ but was validated against experimental ion-acetate binding free energies using an ECC charge scaling parameter of *f* = 0.80.^20,55,59^ To test the effect of ECC charge scaling on the other force fields listed in Table 1, we use an ECC charge scaling parameter of *f* = 0.80 with published 12-6 Lennard-Jones parameters (Table S1).

**Table 1:** Force Fields Are for Alkaline Earth Metal Ions

force field	water model	experimental properties^a^	alkaline earth metal ions	ECC tested?	acetate binding tested?
Li et al.^b^	TIP3P	Δ*G*_solv_, CN	Mg^2+^, Ca^2+^, Sr^2+^, Ba^2+^	No	No
	SPC/E	Δ*G*_solv_, CN		No	Yes^c^
Mamatkulov and Schwierz^d^	TIP3P	Δ*G*_solv_, *a*_cc_, τ	Mg^2+^, Ca^2+^, Sr^2+^, Ba^2+^	No	No
Mendes de Oliveira et al.^e^	TIP4P	Δ*S*(*Q*)	Mg^2+^, Ca^2+^	Yes^f^	No
	SPC/E	Δ*S*(*Q*)		Yes^f^	Yes^c^

a Experimental properties used to parametrize each force field, including free energy of solvation (Δ*G*_solv_), ion–water coordination number (CN), derivative of the activity coefficient (*a*_cc_), water residence time (τ), and neutron scattering difference structure factors (*ΔS*(*Q*)).

b TIP3P compromise (CM) parameters from ref 33.

c Tested for Mg^2+^ and Ca^2+^ using the SPC/E CM parameters from ref 33.^20^

d Reference 32.

e Reference 20.

f References 59 and^55^.

### Simulated Adhesion Force Curves

Simulated adhesion force curves may be calculated by applying a harmonic potential with a constant pull rate to a bound divalent metal ion. By pulling the harmonic potential along the *z*-coordinate, the forces acting on the divalent metal ion as a function of the distance from the MUA monolayer may be sampled. Here, a harmonic potential with a force constant of 5,000 kJ/mol·nm^2^ and a constant pull rate of 0.00002 nm/ps was applied to the *z*-component of the vector between the center of mass (COM) of MUA and a bound divalent metal ion from the last step of a well-equilibrated production simulation. The forces sampled during each constant pull rate simulation are plotted as a function of the distance. This data are analyzed using bin averaging with a bin size of 500 data points, where each data point is 10 ps of constant pull rate simulation time. The calculated adhesion force is defined as the difference between the largest magnitude average force and the average force at large distances, which is approximately 0.0 nN. The average adhesion force measured by an AFM tip is calculated as the mean adhesion force from all bound divalent metal ions within the surface area of the AFM tip, which is 3 to 6 nm^2^.^24^ Based on the Cartesian coordinates from the last step of the production simulation, constant pull rate simulations were performed for each bound divalent metal ion within an approximate 2.4 nm x 2.4 nm square centered on the MUA monolayer. In total, the average adhesion force of Mg^2+^, Ca^2+^, Sr^2+^, and Ba^2+^ was calculated from constant pull rate simulations of 10, 14, 16, and 18 ions, respectively. Simulated adhesion force curves were calculated using the Li et al. force field for alkaline earth metal ions without ECC charge scaling (see Table 1).

### Potential of Mean Force (PMF) Binding Free Energy

Umbrella sampling is used to calculate the potential of mean force (PMF) from which the binding free energy may be calculated.^63^ Here, the PMF reaction coordinate is defined as the *z*-component of the vector from the COM of the MUA monolayer to the divalent metal ion. A harmonic potential with a force constant of 5,000 kJ/mol·nm^2^ was applied to about 10 divalent metal ions to sample the PMF along the reaction coordinate. Divalent metal ions were chosen based on their initial positions along the reaction coordinate from the last step of a well-equilibrated production simulation. The weighted histogram analysis method (WHAM)^64^ implemented in GROMACS 2024 was used to calculate the PMF based on histogram data from a series of umbrella sampling simulations using the Bayesian bootstrap method and 200 bootstraps. The binding free energy is defined as the difference between a minimum along the PMF curve and the PMF at large distances, which is approximately 0.0 kJ/mol. PMF curves were calculated using the Li et al. force field for alkaline earth metal ions without ECC charge scaling (see Table 1).

### Langmuir Isotherm Binding Free Energy

Binding free energy from PMF calculations and average adhesion forces from constant pull rate simulations assume that the relative binding strength is dominated by site-specific interactions between the divalent metal ion and MUA monolayer. Alternatively, divalent metal ion binding may be driven by a collective ion mechanism, in which the distribution between bound and unbound ions determines the relative binding free energy. The Langmuir isotherm^65,66^ quantifies this ion distribution as the standard equilibrium adsorption constant (*K*_0_),

$$K_{o}=\frac{q_{e}}{\left(q_{m}-q_{e}\right)}\cdot \frac{C_{o}}{C_{e}}$$ 2

where *q*_e_ and *q*_m_ are the equilibrium and maximum absorption capacities of the absorbent and *C*_e_ and *C*_0_ are the equilibrium and standard state concentrations of absorbate, respectively. Absorption capacities are reported in units of milligrams of absorbate per gram of absorbent, and concentrations are reported in units of moles of absorbate per L of solution. Here, *q*_e_ is calculated as the average number of divalent metal ions bound to the MUA monolayer from the last 100 ns of the production simulation. *q*_e_ is calculated by assuming all divalent metal ions are bound to the MUA monolayer because there are fewer divalent metal ions than the number of binding sites on the simulated MUA monolayer. *C*_e_ is calculated as the average molar concentration of unbound divalent metal ions from the last 100 ns of the production simulation. *C*_0_ is defined as the standard state molar concentration of 1 mol/L. The calculated *K*_0_ value is then used to calculate the standard binding free energy (*ΔG°*),

3 $$\Delta G^{{}^{\circ}}=-RT\;\ln K_{o}$$

where *R* is the ideal gas constant and *T* is the production simulation temperature in K. Langmuir isotherm binding free energies were calculated using the alkaline earth metal ion force fields listed in Table 1.

### Experimental AFM Binding Free Energy

Simulated results from this work are compared to experimental data from AFM experiments by Rios-Carvajal et al. (Table S4).^24^ Their work reports average adhesion forces for replicate AFM measurements using a carboxylate-functionalized AFM tip and a carboxylate-functionalized surface in aqueous solutions of alkaline earth metal ion chloride salts. These AFM adhesion forces may be directly compared to the average adhesion forces calculated using constant pull simulations. For comparison with binding free energies calculated using PMF curves or the Langmuir isotherm, the AFM adhesion force (*F*_AD_) is used to calculate binding free energies (*ΔG*_bind_),^16^

$$\Delta G_{\mathrm{bind}}=\frac{F_{\mathrm{AD}}\cdot L}{k_{B}T}$$ 4

where *L* is the length of the binding group, *k*_B_ the Boltzmann’s constant, and *T* is temperature in K. This approach was used to convert experimental AFM adhesion forces to binding free energies of DNA adsorbed to a MUA monolayer in the presence of Mg^2+^, Ni^2+^, or Co^2+^ ions.^16^ In our previous work, *L* was defined as the distance between DNA base pairs (*L* = 3.32 Å) because this was the distance necessary to peel the adsorbed DNA from the MUA monolayer in a quasi-equilibrium process.^16^ Analogously, *L* is defined here as the distance between the oxygen atoms of the carboxylate group^24,67^ (*L* = 2.2 Å) because this is the distance that must be traversed to remove one carboxylate group from an ion bridge complex formed between the carboxylate-functionalized AFM tip and the MUA monolayer. For a direct comparison to our simulation data, *ΔG*_bind_ is reported per carboxylate group on the AFM tip (Table S4). This value is calculated using the approximate AFM tip coverage of deprotonated carboxylate groups (12–27 carboxylate groups) reported by Rios-Carvajal et al.^24^ An alternative definition of *L* is the size of the alkaline earth metal ion. Depending on how likely an ion remains fully hydrated, Rios-Carvajal et al. used the hydrated radius (about 4 Å) or the ionic radius (about 1 Å) of each alkaline earth metal ion as the distance of interaction to estimate the Coulombic force necessary to break one ion-carboxylate interaction within an ion bridge complex.^24^ Using this definition of *L* would scale the magnitude of *ΔG*_bind_, but the qualitative trend among alkaline earth metal ions would remain the same. Here, we use *L* = 2.2 Å because this definition is analogous to that in our previous work.

An alternative approach to calculating binding free energy from AFM forces is proposed by Friddle et al., where the binding free energy is proportional to the square of the equilibrium force and, within the equilibrium limit, the equilibrium force is equal to the adhesion force.^68,69^ Although we cannot apply this approach to the AFM results reported by Rios-Carvajal et al., because the loading rate and the dependence of the adhesion force on the loading rate were not reported, we applied this approach to our previous work that reported an adhesion force of 78.4 ± 9.8 pN for pulling DNA from MUA in 5 mM Ni^2+^ solution using a cantilever force constant of approximately 0.09 N/m within the equilibrium regime (i.e., the adhesion force is independent of the loading rate).^16^ The approach by Friddle et al. predicts a binding free energy of 20.6 ± 5.4 kJ/mol compared to 15.7 ± 2.0 kJ/mol calculated using the equation shown above. The agreement between these values calculated from AFM results previously reported for a DNA-MUA system provides evidence of the reliability of our approach for converting AFM adhesion forces to binding free energies.

## Results and Discussion

To investigate the interplay of local site-specific interactions and larger-scale collective ion binding, we invoke three different approaches to quantifying the strength of alkaline earth metal ion binding at the aqueous interface of a deprotonated MUA monolayer. The first two approaches, simulated adhesion force curves and PMF binding free energies, are site-specific; they sample forces acting on specific atoms and synthesize that data to estimate experimentally observable properties. The third approach, binding free energies from the Langmuir isotherm, uses the equilibrium distribution of ions binding to the monolayer surface to calculate the binding free energy. Analysis of these approaches to reproduce experimental AFM results provides insight into the significance of site-specific versus collective ion binding within the context of interfacial divalent metal ion binding to anionic carboxylate-terminated monolayers in aqueous solution.

### Site-Specific Binding

Divalent metal ions may adsorb to anionic functionalized surfaces through a site-specific binding mechanism, where individual ion binding interactions drive the overall binding strength. Constant pull simulations may be used to simulate the adhesion force curves for individual bound divalent metal ions. The calculated force curve for a Mg^2+^ ion directly bound to the MUA monolayer (Figure 2a) resembles the AFM force curves reported by Rios-Carvajal et al.^24^ Average adhesion forces were calculated as the mean adhesion force for all bound divalent metal ions within the approximate surface area of the AFM tip (Figure 2b). The error bars reported in Figure 2b indicate the standard deviation of adhesion forces measured from a single MD simulation. We expect replicate simulations to reduce this standard deviation, but additional simulations were not performed because simulated adhesion forces do not reproduce the experimental trend from AFM. Although the calculated average adhesion force has the same order of magnitude (∼1–2 nN) as the experimental AFM results (∼0.5–1.5 nN), the trend among alkaline earth metal ions is reversed. Simulated average adhesion forces predict that Mg^2+^ has a stronger interaction with the carboxylate interface than does Ca^2+^. This trend is consistent with vibrational spectroscopy^20^ and potentiometric titrations^21^ of dilute aqueous solutions of acetate, which are inherently site-specific because the divalent metal ions are bound in a one-to-one ratio with acetate ions in dilute aqueous solutions. However, interfacial AFM experiments reveal that Mg^2+^ ions induce a weaker adhesion force than Ca^2+^ ions.^24^ This discrepancy indicates that the site-specific nature of calculating average adhesion forces by pulling individual divalent metal ions from the carboxylate monolayer does not accurately capture the important factors that determine the relative strengths of ion-carboxylate adsorption at an aqueous interface. Our analysis suggests that simulated adhesion force curves calculated for an ion-monolayer system more closely align with individual ion-carboxylate complexes measured in dilute aqueous solutions rather than interfacial AFM results.

**Figure 2:**
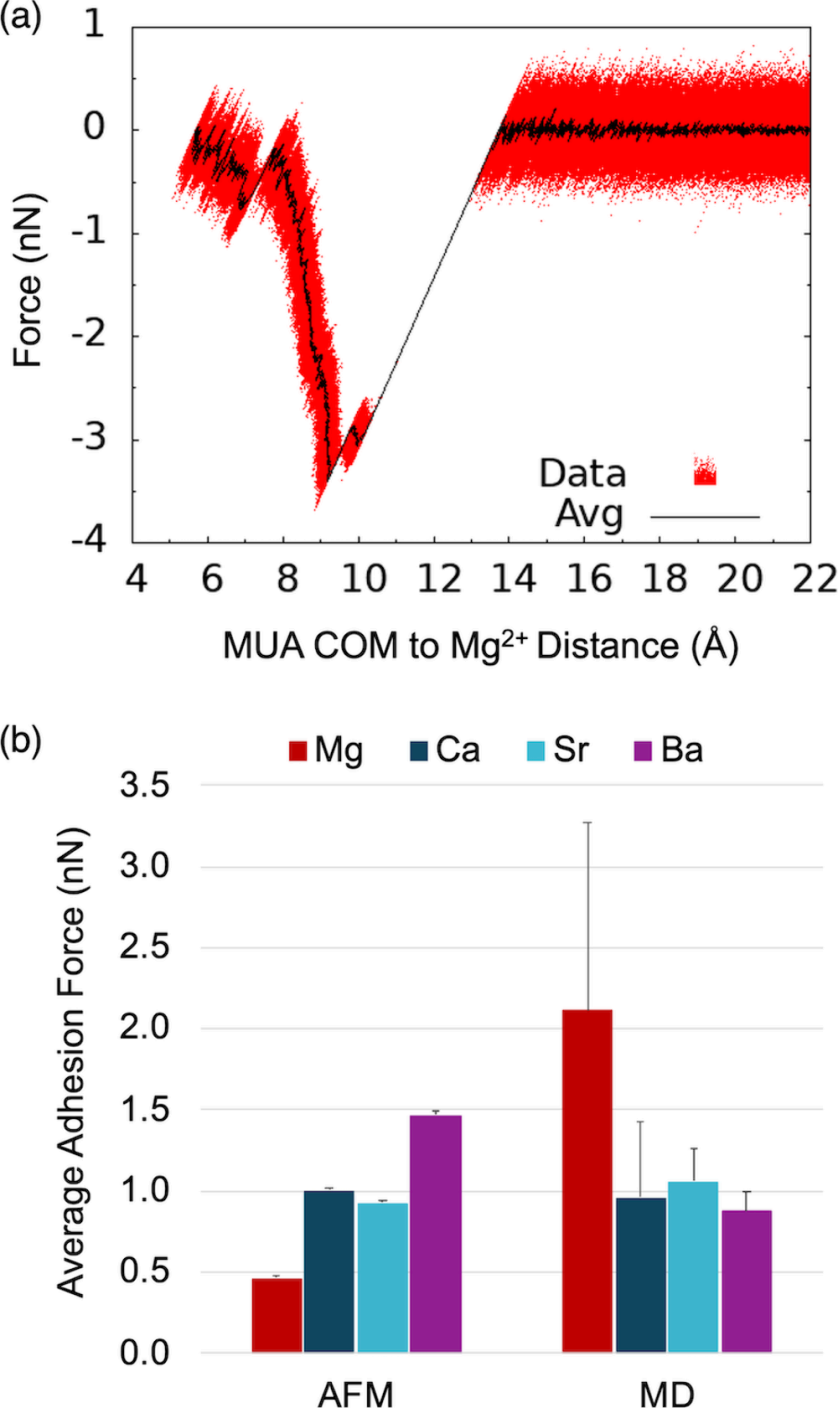
(a) Simulated adhesion force curve of a Mg^2+^ ion directly bound to the MUA monolayer. Raw data (Data) and the mean value of every 500 data points (Avg) are shown. (b) Average adhesion force from AFM experiments and molecular dynamics (MD) simulations. Error bars for AFM data are the standard deviation of 900 AFM force curves. Error bars for MD data are the standard deviation of 10–18 simulated force curves (one for each divalent metal ion within the approximate surface area of the AFM tip). AFM data were reproduced from ref 24. Copyright 2019 American Chemical Society.

An alternative site-specific approach is to use umbrella sampling to calculate the PMF free energy curve. Instead of pulling ions from the monolayer surface, this approach samples ion configurations at different distances from the surface. These configurations are then used to calculate a PMF free energy curve as a function of the distance from the monolayer surface. The PMF free energy curves calculated for each alkaline earth metal ion reveal that the ions bind near 6 Å from the center of mass of the MUA monolayer (Figure 3a). As this distance increases, the free energy approaches zero, implying that the ion is unbound from the MUA monolayer. The difference in free energy from the bound state near 6 Å and the unbound state at long distances estimates the binding free energy of the ion to the MUA monolayer. These binding free energies range from 30 to 170 kJ/mol. For comparison to experimental AFM results, the binding free energies are divided by the average number of MUA ligands bound per divalent metal ion (4.7 for Mg^2+^ and 4.4 for Ca^2+^, Sr^2+^, and Ba^2+^). The PMF binding free energies overestimate the AFM results^24^ by 1 order of magnitude (Figure 3b). For comparison, PMF free energy curves calculated using *ab initio* MD simulations also overestimate alkaline earth metal ion binding to acetate in aqueous solution.^23^ Our classical MD simulations of an ion-monolayer system reproduce the qualitative experimental trend among Mg^2+^, Ca^2+^, and Sr^2+^, but they underestimate the binding free energy of Ba^2+^ relative to Ca^2+^. Using ECC charge scaling inverts the relative binding free energy between Mg^2+^ and Ca^2+^ (Figure S2), which qualitatively disagrees with experimental AFM results.^24^ ECC charge scaling reduces Ca^2+^ PMF binding free energy by about 100 kJ/mol but increases the Mg^2+^ PMF binding free energy by more than 200 kJ/mol. Given that ECC charge scaling is expected to reduce the strength of the local ion-carboxylate interaction, this result suggests that more complicated factors are at play than the simple ion-carboxylate binding strength. Taken together, our results suggest that free energies calculated from local ion configurations sampled along the reaction coordinate do not reliably reproduce the qualitative trend or the quantitative magnitude of interfacial AFM results.

**Figure 3:**
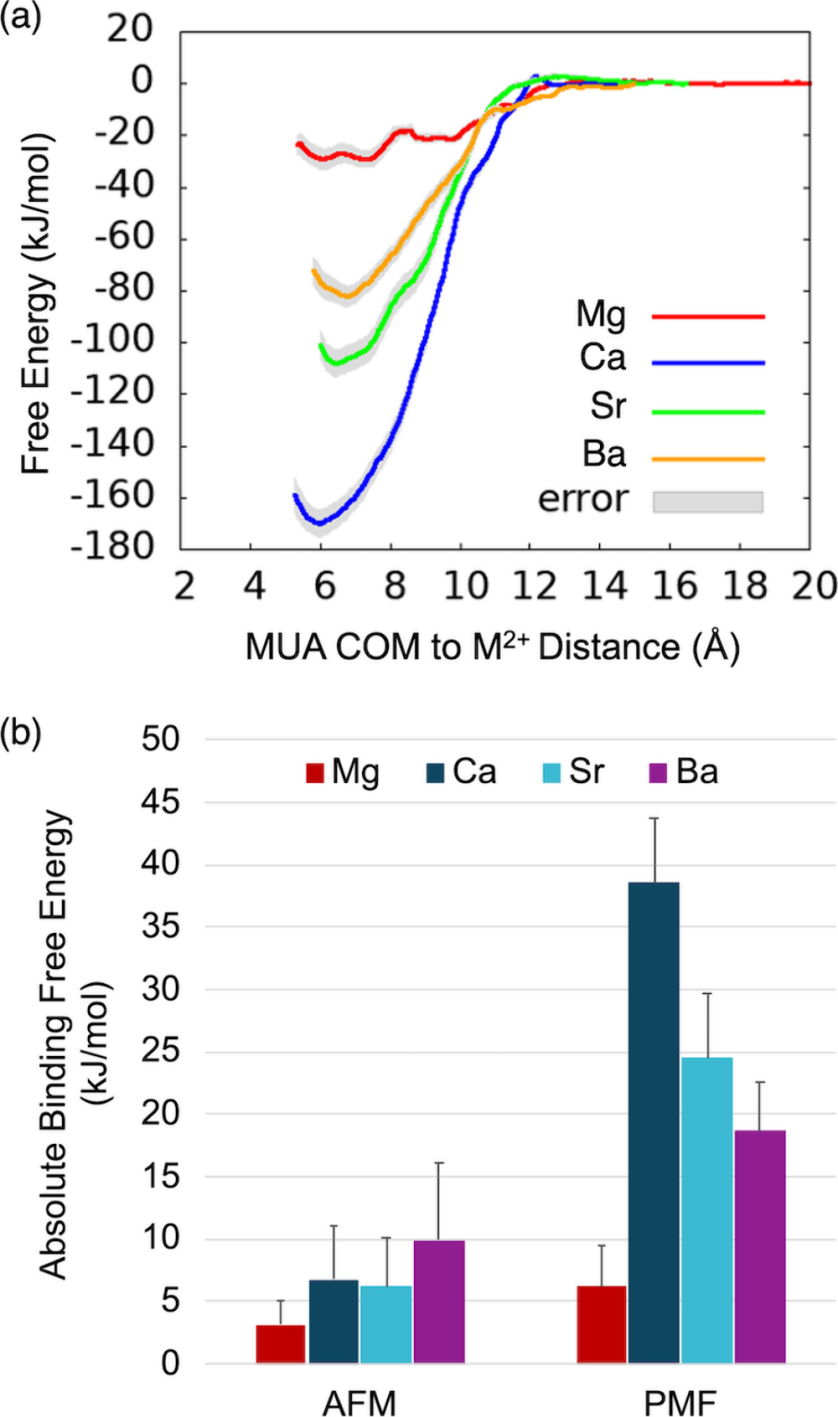
(a) PMF free energy curves for binding of M^2+^ (M = Mg, Ca, Sr, Ba) to a deprotonated MUA monolayer. The reaction coordinate is the distance between the center of mass (COM) of the MUA monolayer and M^2+^. (b) Absolute value of the binding free energy per bound MUA ligand calculated from AFM average adhesion forces and PMF free energy curves. AFM data were adapted from ref 24. Copyright 2019 American Chemical Society.

Although PMF binding free energies are a well-established computational approach and adhesion force curves can easily be calculated using constant pull rate simulations, neither of these site-specific approaches reproduces the interfacial AFM results for a simple ion-monolayer system. These site-specific approaches use the forces acting on individual divalent metal ions to estimate an experimentally observable property. In other words, these computational approaches assume that the adhesion force and binding free energy depend on the site-specific interactions of individual divalent metal ions with the MUA monolayer. Because neither approach successfully reproduces both the qualitative trend and the quantitative magnitude of AFM results, we conclude that local, site-specific interactions modeled using an ion-monolayer system do not accurately capture the physical interactions that drive divalent metal ion binding at the aqueous interface of anionic functionalized surfaces.

### Collective Ion Binding

Given that site-specific approaches do not capture the physically relevant behavior of divalent metal ions at an aqueous carboxylate monolayer interface, we aimed to quantify ion adsorption using an approach that considers the overall distribution of ions across the interface. The Langmuir isotherm^65,66^ quantifies the equilibrium distribution of adsorbed species, which can be used to calculate a binding free energy (*ΔG°*). Unlike site-specific approaches, this collective ion binding approach uses equilibrium simulations to predict the average number of divalent metal ions bound to the MUA monolayer, which is used as input for the Langmuir isotherm.

Using the Langmuir isotherm, several alkaline earth metal ion force fields were tested for their ability to reproduce the experimental AFM trend that Mg^2+^ induces weaker ion adsorption than Ca^2+^ (Figure 4). The Li et al. force field with and without ECC charge scaling and the Mamatkulov and Schwierz and Mendes de Oliveira et al. force fields with ECC charge scaling reproduce the experimental AFM trend (i.e., Mg^2+^ binding is weaker than Ca^2+^ binding). The Mendes de Oliveira et al. force field, which was parametrized to reproduce ion-acetate binding free energies from dilute solutions of acetate, underestimates the Mg^2+^ binding free energy and overestimates the Ca^2+^ binding free energy. The Mamatkulov and Schwierz force field with ECC charge scaling overestimates both the Mg^2+^ and Ca^2+^ binding free energies. The Li et al. force field with and without ECC charge scaling reproduce the average AFM binding free energy values within 8 and 5 kJ/mol, respectively. Accounting for the standard deviation of the experimental AFM binding free energies, these differences are reduced to 3 and 0.5 kJ/mol, respectively. These results demonstrate that unscaled alkaline earth metal ion point charges combined with the 12-6 Lennard-Jones parameters from Li et al.^33^ quantitatively reproduce experimental binding free energies to a carboxylate-functionalized surface. Contrary to previous simulations of ion-carboxylate binding in a dilute aqueous solution,^20^ ECC charge scaling does not improve the accuracy of the equilibrium MD simulations of alkaline earth metal ion adsorption to a carboxylate-terminated monolayer reported here. In general, reducing the strength of individual ion-carboxylate interactions by applying ECC charge scaling increases the number of bound ions at the carboxylate interface, which overestimates the binding free energy calculated by using the Langmuir isotherm. This result suggests that reducing the electrostatic attraction between individual alkaline earth metal ions and carboxylate anions increases the occupancy of the MUA monolayer by binding more divalent metal ions.

**Figure 4:**
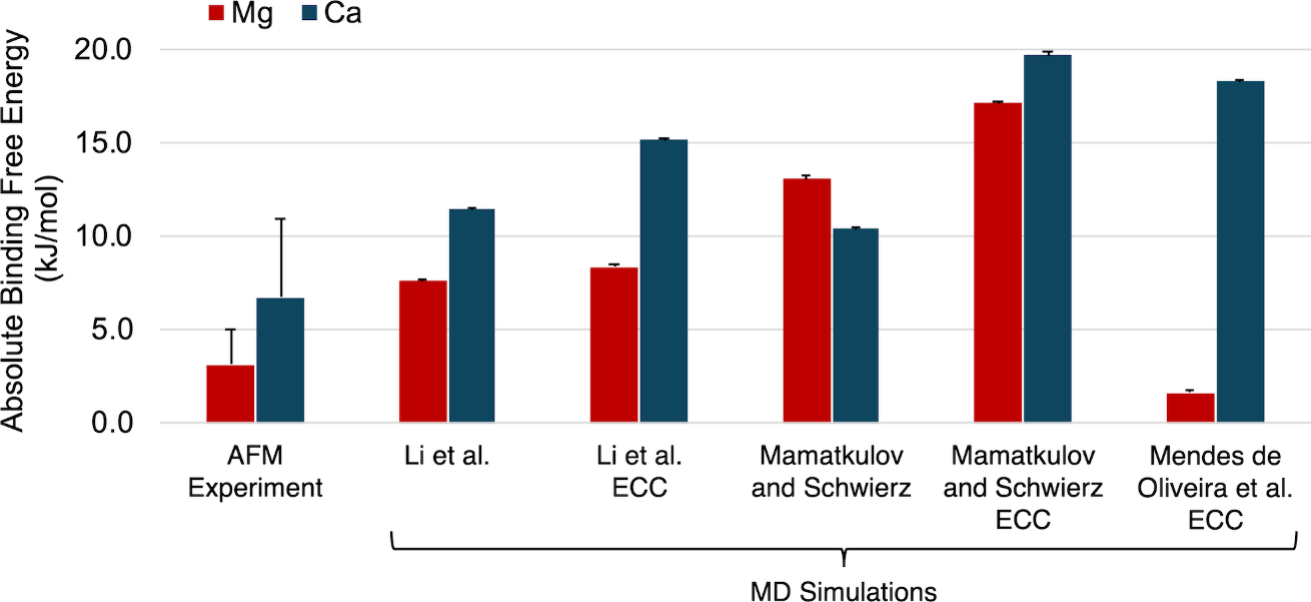
Absolute binding free energies (in kJ/mol) of Mg^2+^ or Ca^2+^ to MUA calculated using adhesion forces from AFM experiments or equilibrium ion distributions from classical MD simulations with different alkaline earth metal ion force field parameters (see Table 1). Error bars for AFM data represent the maximum spread in *ΔG*_bind_ per carboxylate group. Error bars for MD simulation data are the deviation in *ΔG°* due to the standard deviation of the number of alkaline earth metal ions bound in each MD simulation. AFM data were adapted from ref 24. Copyright 2019 American Chemical Society.

The Li et al. force field without ECC charge scaling quantitatively reproduces interfacial AFM results; therefore, it was used to calculate *ΔG°* for all four alkaline earth metal ions (Figure 5). The simulations reported here predict *ΔG°* values (7–12 kJ/mol) on the same order of magnitude as the AFM results (3–16 kJ/mol). Importantly, the Langmuir isotherm binding free energies reproduce the trend among alkaline earth metal ions reported by AFM results (i.e., Mg^2+^ < Sr^2+^ < Ca^2+^ < Ba^2+^), with the exception that Ca^2+^ and Ba^2+^ have similar binding free energies. Although the calculated *ΔG°* values overestimate the binding free energy for Mg^2+^, Ca^2+^, and Sr^2+^, the relative change between ions is reproduced within 1 kJ/mol. The Ba^2+^ binding free energy is overestimated relative to the average AFM value, but is within the experimental error. The difference in Ba^2+^ binding free energy relative to Ca^2+^ is underestimated compared to the AFM results by 3 kJ/mol. This discrepancy may be due to most divalent metal ions binding to the MUA monolayer at equilibrium. Equilibrium MD simulations at lower divalent metal ion concentrations reveal concentration-dependent ion adsorption (Table S2). This result is consistent with theoretical models^29,37^ and experiments^5,40,42,70^ that demonstrate concentration-dependent ion effects. Given that ion adsorption peaks at intermediate concentrations,^37,40^ repeating these MD simulations at different divalent metal ion concentrations may improve quantitative agreement with the AFM results by revealing the ion-specific effects of concentration on ion adsorption. Overall, the Li et al. force field without ECC charge scaling and the Langmuir isotherm approach to calculating binding free energy accurately model alkaline earth metal ion binding to a carboxylate-functionalized surface.

**Figure 5:**
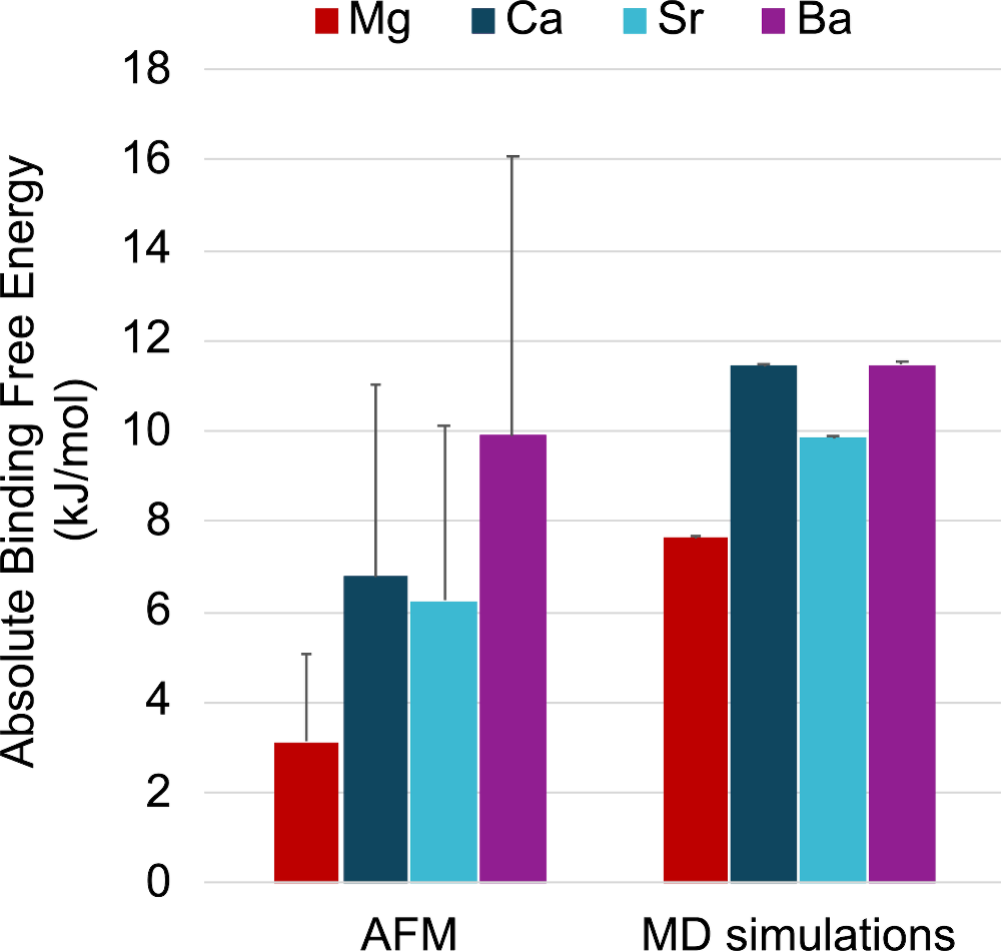
Absolute binding free energies (in kJ/mol) of Mg^2+^, Ca^2+^, Sr^2+^, or Ba^2+^ to a carboxylate-terminated alkanethiol monolayer calculated using adhesion forces from AFM experiments or equilibrium ion distributions from classical molecular dynamics (MD) simulations with the Li et al. force field for alkaline earth metal ions without the ECC charge scaling (see Table 1). Error bars for AFM data represent the maximum spread in *ΔG*_bind_ per carboxylate group. Error bars for MD simulation data are the deviation in *ΔG°* due to the standard deviation of the number of alkaline earth metal ions bound in each MD simulation. AFM data adapted from ref 24. Copyright 2019 American Chemical Society.

Comparison of the site-specific and collective ion binding approaches to quantifying the strength of alkaline earth metal ion binding to the aqueous interface of an anionic carboxylate-terminate monolayer suggests that the site-specific approaches qualitatively agree with experimental and computational results for dilute solutions of carboxylate anions, whereas the collective ion approach reproduces interfacial data from AFM experiments. Therefore, classical force fields parametrized to reproduce local ion properties, such as ion hydration free energy and ion–water oxygen distances, can accurately model divalent metal ion adsorption at the aqueous interface of anionic carboxylate monolayers. However, the analysis of interfacial ion adsorption modeled using an ion-monolayer system must consider the larger-scale distribution of bound and unbound ions to reproduce interfacial experimental results. Our MD simulations demonstrate that site-specific ion-carboxylate interactions alone do not accurately capture the physical interactions that govern overall divalent metal ion adsorption. Agreement between the interfacial AFM results and the collective ion binding approach (i.e., the Langmuir isotherm) does not imply that site-specific interactions are unimportant. Rather, our results suggest that the overall distribution of divalent metal ions, which may be influenced by local ion-carboxylate interactions, dominates the ion adsorption process. Given the agreement between interfacial AFM data and our MD simulations using the Langmuir isotherm, we aim to understand the origin of the ion-specific effects of this interfacial process.

### Ion-Specific Effects

Experiments demonstrate that different alkaline earth metal ions exhibit different binding properties to an anionic carboxylate monolayer.^24,25^ Here, we use the Li et al. force field for alkaline earth metal ions (see Table 1) without ECC charge scaling to investigate the origin of these ion-specific effects.

The radial distribution function (RDF) between the carboxylate carbon atom and the divalent metal ion reveals that all alkaline earth metal ions form direct contacts with the carboxylate group of the MUA monolayer (Figure 6a). The first and second peaks in the RDF represent bidentate and monodentate direct binding, respectively (Figure 6b). Mg^2+^ exhibits indirect or solvent-shared binding, as demonstrated by the third RDF peak between 3.8 and 5.9 Å. This peak encompasses two types of indirect binding indicated by the slight dip in the RDF curve at 4.5 Å. The majority of indirectly bound Mg^2+^ ions (30.6 of 37.7 ions) are within 3.8–4.5 Å and form hydrogen bonds with both oxygen atoms of at least one MUA ligand, whereas a smaller portion (7.1 of 37.7 ions) are within 4.5–5.9 Å and form only one hydrogen bond per MUA ligand. The RDFs shown in Figure 6a were used to define distance cutoffs for bidentate direct binding, direct binding (i.e., bidentate and monodentate), and total binding (i.e., direct and indirect binding) (Table S5). Based on these distance cutoffs, the average number of divalent metal ions engaged in different binding motifs were calculated, including simultaneous binding of different types (Figure 6c).

**Figure 6:**
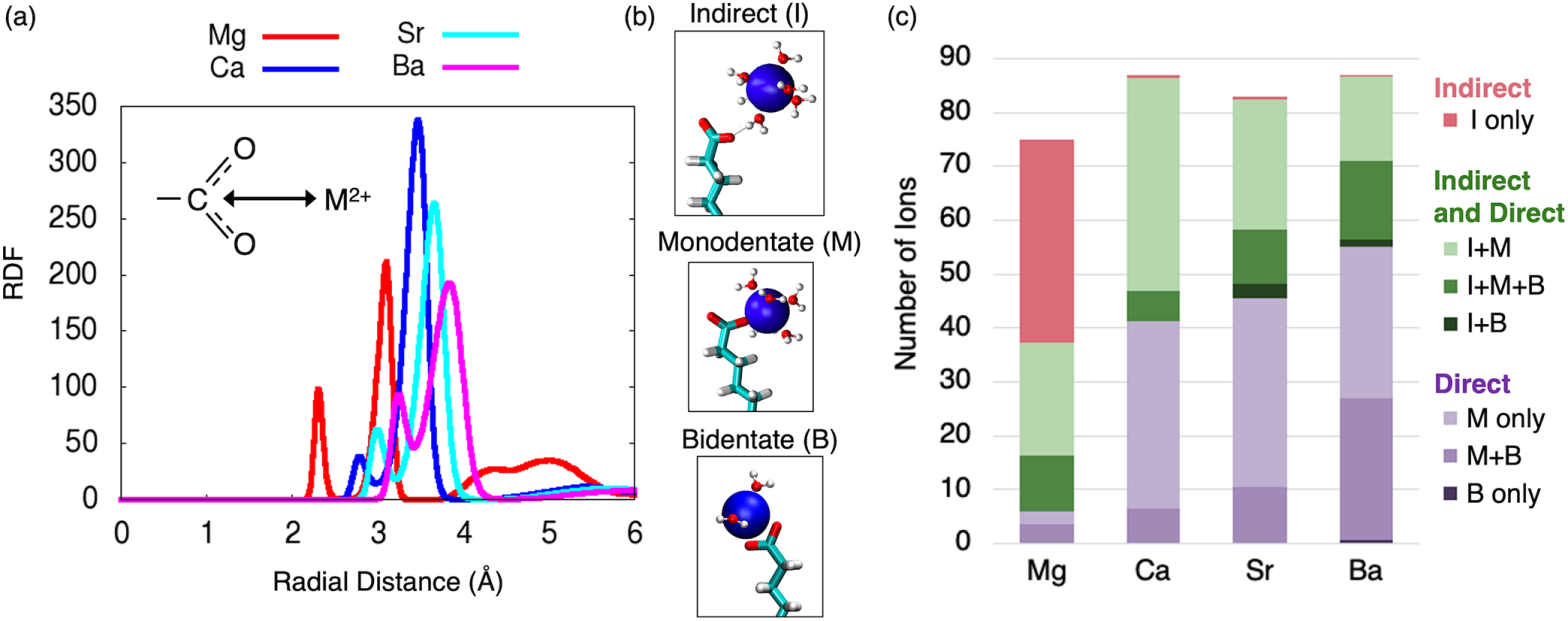
(a) Radial distribution function (RDF) from the carboxylate carbon atom of MUA to each divalent metal ion. Inset shows the distance used to calculate the RDF. (b) Molecular structures of indirect binding (I), monodentate direct binding (M), and bidentate direct binding (B). The divalent metal ion is shown as a blue sphere. The metal ion’s first solation shell is shown in the ball and stick representation. The MUA ligand is shown in the licorice representation, where the O, C, and H atoms are red, cyan, and white, respectively. Other MUA ligands, water molecules, and ions are omitted for clarity. (c) Average number of divalent metal ions bound to the MUA monolayer categorized by binding motif. The “+” symbol indicates simultaneous binding of different types.

Similar to our previous work modeling alkaline earth metal ion binding to double-strand DNA,^71^ the tightly held first solvation shell of Mg^2+^ leads to significant indirect binding, whereas the larger alkaline earth metal ions are more likely to form direct contacts due to their more flexible first solvation shells (Figure 6c). This result is consistent with interfacial spectroscopy experiments that suggest Mg^2+^ predominately forms solvent shared ion pairs,^17,72^ whereas Ca^2+^ ions may form bidentate or bridging complexes as reported by vibrational sum frequency generation spectroscopy^72^ or a combination of solvent-shared, monodentate direct, and bidentate direct contacts as reported by IRRAS.^17^

Our MD simulations predict that the MUA monolayer induces a nearly equal distribution of direct and indirect binding for Mg^2+^ (Figure 6c). Most Mg^2+^ ions that form direct contacts also form indirect contacts. Of these ions, more than half form monodentate direct contacts only, whereas the rest form bidentate and monodentate direct contacts. Few Mg^2+^ ions form direct contacts only; these ions form either monodentate direct contacts only or bidentate and monodentate direct contacts.

The Ca^2+^, Sr^2+^, and Ba^2+^ ions bind primarily through direct contacts to the MUA carboxylate group (Figure 6c). Ca^2+^ is equally likely to form simultaneous indirect and direct contacts as it is to form direct contacts only. In either case, Ca^2+^ prefers monodentate direct contacts over bidentate direct contacts. As the divalent metal ion size increases, the portion of bound ions that form bidentate contacts increases, and the portion of bound ions that form indirect contacts decreases. Ca^2+^, Sr^2+^, and Ba^2+^ ions rarely form bidentate direct contacts only. In general, these ions prefer to form monodentate direct contacts only or simultaneous monodentate and bidentate direct contacts; in either case, these ions may or may not form indirect contacts along with the direct contacts.

These ion-specific binding motifs are consistent with the ion distribution along the height of the simulation box. Number density plots reveal two layers of Mg^2+^ ions; one embedded within the carboxylate groups and another above the carboxylate oxygen atoms (Figure S3). In contrast, the majority of the larger alkaline earth metal ions are located within the distribution of carboxylate oxygen atoms. The position of the maximum divalent metal ion number density aligns with an excess positive charge density and an inversion of the electrostatic potential (Figure S4). This charge inversion is consistent with theoretical predictions from a model that explicitly accounts for ion correlation and excluded volume effects.^37,38^ However, additional work is necessary to investigate the magnitude of the electrostatic potential as a function of the divalent ion concentration and other system parameters.

The prevalence of simultaneous binding of different types suggests that multiple MUA ligands bind to a single divalent metal ion. The average number of MUA ligands bound per bound ion was calculated for each alkaline earth metal ion and categorized by a binding motif using the RDF distance cutoffs for bidentate and direct binding (Table 2). For comparison, the same data were calculated for a Na^+^ reference simulation that did not contain divalent metal ions. This reference simulation predicts that, on average, three MUA ligands bind Na^+^ ions through direct contacts, which agrees with a previous MD simulation.^30^ Similar to Na^+^, Mg^2+^ ions bind about three MUA ligands through direct contacts. On average, the larger alkaline earth metal ions bind up to one additional MUA ligand through direct contacts. This average value increases with the ion size and correlates with an increase in the portion of bound ions that form bidentate direct contacts (Figure 6c). On average, one or two MUA ligands that form a direct contact do so in a bidentate fashion. Mg^2+^ ions form a one-to-one ratio with bidentate MUA ligands, whereas Ca^2+^, Sr^2+^, and Ba^2+^ ions bind one or two MUA ligands in a bidentate motif. In total, the alkaline earth metal ions bind on average four to five MUA ligands simultaneously, which is similar to the Na^+^ reference simulation. Overall, the total number of MUA ligands bound per ion is similar among alkaline earth metal ions, but the portion of bound MUA ligands that form direct contacts increases with an increase in ion size. This increase correlates with a larger portion of bound ions that form bidentate direct contacts.

**Table 2:** Physical properties of the MUA monolayer in the presence of different metal ions

	number of MUA ligands per ion^a^				
ion	bidentate	direct	total	MUA intermolecular O–O distance (Å)^b^	MUA tilt angle (°)^c^
Na^+^	1.2	3.0	4.6	3.5	25.1 ± 11.6
Mg^2+^	1.0	2.9	4.7	2.8	13.5 ± 11.8
Ca^2+^	1.2	3.5	4.4	3.0	17.4 ± 12.6
Sr^2+^	1.2	3.7	4.4	3.2	17.6 ± 10.4
Ba^2+^	1.2	3.9	4.4	3.4	16.7 ± 13.0

a Mean number of MUA ligands bound per metal ion with at least one bidentate direct contact (bidentate), at least one bidentate or monodentate direct contact (direct), and at least one direct or indirect contact (total). Mean values vary by ±0.02 due to standard deviation of time-averaged data.

b Radial distance of the maximum of the second RDF peak between MUA carboxylate oxygen atoms (Figure S5).

c Mean and standard deviation of the MUA tilt angle, defined as the angle between the vector passing through the MUA hydrocarbon chain and the vector normal to the monolayer surface.

Our analysis indicates that as the ion size increases, the alkaline earth metal ions shift their binding motif to form more bidentate direct contacts. However, DFT computed binding energies demonstrate that ion-carboxylate complexes in aqueous solution exhibit weaker interactions as the ion size increases.^22,23^ Therefore, the strength of the ion-carboxylate interaction cannot explain the size-dependent trends in the binding motif. Furthermore, the binding free energies predicted by the Langmuir isotherm (Figure 5) do not correlate with ion size, the number of direct contacts formed per ion (Figure 6c), or the portion of ions that form bidentate direct contacts. Given that this collective ion binding approach is dependent on the equilibrium distribution of bound to unbound divalent metal ions, we investigate how efficiently the alkaline earth metal ions occupy the carboxylate-terminated monolayer surface. Mg^2+^ is unique among the divalent metal ions studied because it exhibits significant solvent shared ion pairs formed through indirect binding only, whereas Ca^2+^, Sr^2+^, and Ba^2+^ typically form at least one direct contact to the carboxylate monolayer (Figure 6c). To compare across all four alkaline earth metal ions, we focused our analysis on direct binding only.

When alkaline earth metal ions bind to the MUA monolayer, the spacing among the carboxylate groups may be distorted across the monolayer interface. All simulated systems have an intramolecular MUA O–O distance of 2.2 Å (Figure S5), in agreement with the experimental O–O distance within a single carboxylate group.^24,67^ However, the intermolecular O–O distance between neighboring MUA ligands exhibits a size-dependent trend (Table 2). This distance corresponds to the maximum of the second peak in the RDF of the MUA carboxylate oxygen atoms (Figure S5). The average MUA intermolecular distance is similar for Ba^2+^ ions relative to a Na^+^ reference simulation (i.e., no divalent metal ions), but the smaller alkaline earth metal ions reduce the MUA intermolecular distance by 0.3–0.7 Å. These results suggest that the smaller alkaline earth metal ions attract neighboring MUA carboxylate groups, resulting in more varied carboxylate group spacing within the MUA monolayer (e.g., Figure 7a–f). The MUA oxygen atoms in the presence of Mg^2+^ ions show a relatively close spacing between the second and third peaks and a broader peak near 5 Å (Figure S5). This result is consistent with the smaller ion radius of Mg^2+^ compared to the other alkaline earth metal ions and the more uneven distribution of the MUA carboxylate groups. The shape of the Ca^2+^ MUA carboxylate O–O RDF is most similar to that of Mg^2+^, but the distribution of the O–O distances is less broad. The Sr^2+^ MUA carboxylate O–O RDF exhibits relatively symmetric peaks, which indicates more equal spacing among the carboxylate groups in the presence of Sr^2+^. In contrast, the second and third peaks of the Ba^2+^ MUA carboxylate O–O RDF exhibit shoulders at slightly shorter O–O distances, which overlap with the maxima of the Ca^2+^ RDF. This result suggests that Ba^2+^ ions have more than one optimal spacing among carboxylate groups that leads to more efficient occupancy of the MUA monolayer interface. Our analysis suggests that ion-specific effects of binding free energies calculated using the Langmuir isotherm can be rationalized by the ability of each ion to efficiently occupy the MUA monolayer. This ability to efficiently occupy the monolayer may be influenced by differences in local ion-carboxylate interactions, but these site-specific interactions alone cannot explain the ion-specific effects reported for interfacial alkaline earth metal ion adsorption.

**Figure 7:**
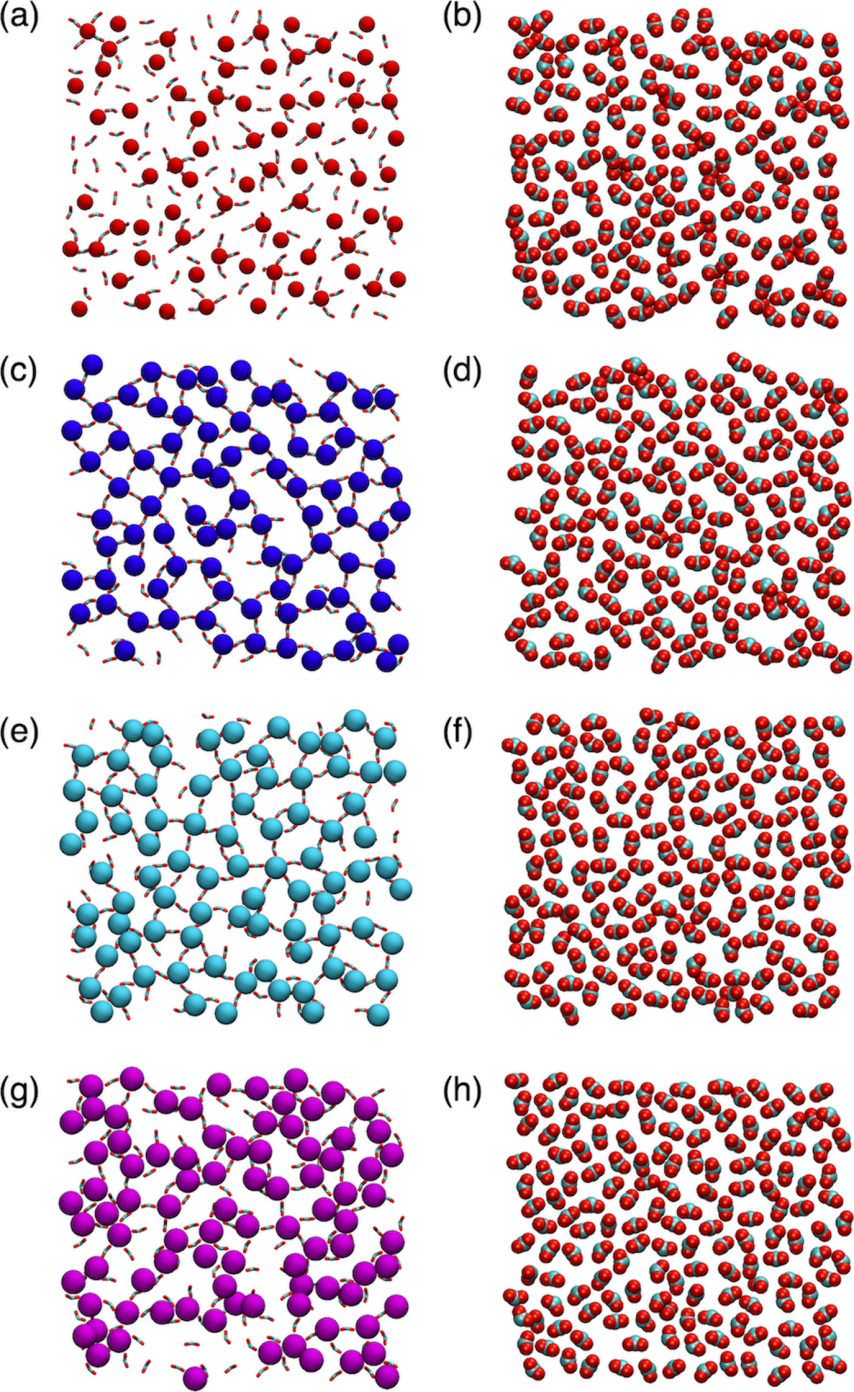
Representative snapshots of alkaline earth metal ions directly bound at the aqueous interface of a deprotonated carboxylate-terminated monolayer. The same structure is shown for each ion: (a, b) Mg^2+^, (c, d) Ca^2+^, (e, f) Sr^2+^, and (g, h) Ba^2+^. The left column (a, c, e, g) shows Mg^2+^, Ca^2+^, Sr^2+^, and Ba^2+^ ions as red, blue, cyan, and magenta spheres, respectively, and the MUA carboxylate groups in the licorice representation. The right column (b, d, f, h) omits the divalent metal ions and shows the carboxylate C and O atoms as cyan and red spheres, respectively. Additional water molecules, ions, and MUA ligand atoms are omitted for clarity.

All alkaline earth metal ions studied here reduce the MUA tilt angle relative to a Na^+^ reference simulation (Table 2). As a result, the divalent metal ions pull the MUA ligands upright to a more vertical orientation (Figure 8). The Mg^2+^ ions induce the most prominent effect. The larger alkaline earth metal ions Ca^2+^, Sr^2+^, and Ba^2+^ exhibit a similar influence on the MUA tilt angle, which is less severe than the effect of Mg^2+^. The distribution of tilt angles among MUA ligands in the monolayer is similar for all divalent metal ions, as indicated by similar standard deviation values reported in Table 2. The strong effect Mg^2+^ has on the MUA tilt angle may be due to the more severe distortion of the carboxylate spacing across the interface exhibited by Mg^2+^ (Figure 7a,b). This result is consistent with experimental evidence that Mg^2+^ distorts the two-dimensional structure of a fatty acid Langmuir monolayer, but Ba^2+^ does not.^43^ However, care must be taken when directly comparing ion-induced structural effects of Langmuir monolayers and alkanethiol monolayers. Alkanethiol monolayers like MUA form a rigid two-dimensional array^47^ on gold surfaces through covalent thiol bonds. This structural constraint is included in the MD simulations presented here, which may influence how divalent metal ions affect the tilt angle and three-dimensional structure of alkanethiol monolayers relative to Langmuir monolayers.

**Figure 8:**
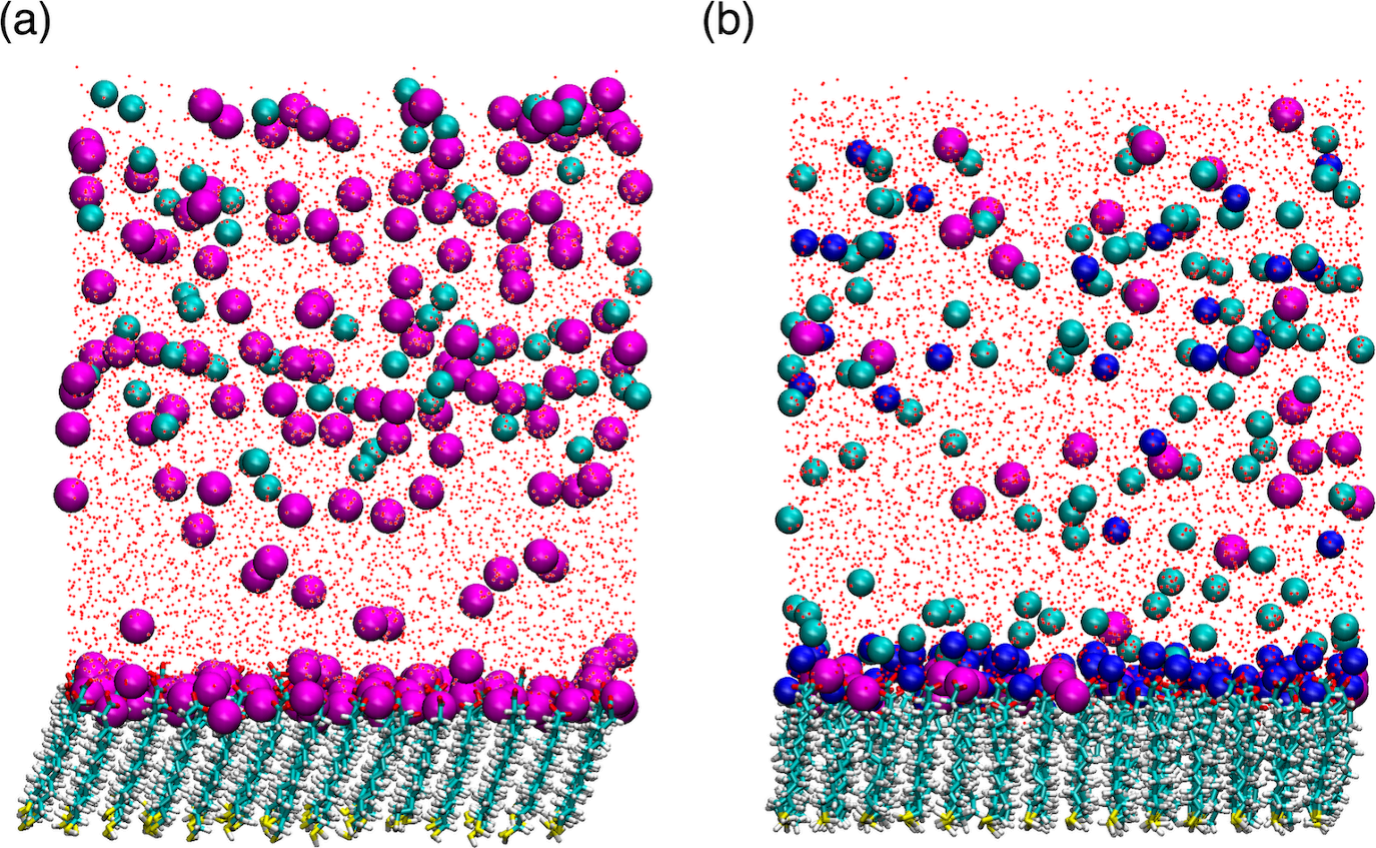
Representative snapshot of the MUA monolayer in the presence of (a) Na^+^ metal ions only and (b) Mg^2+^ and Na^+^ metal ions. Mg^2+^, Na^+^, and Cl^–^ ions are shown as blue, magenta, and cyan spheres, respectively. The water oxygen atoms are shown as red dots. The MUA ligands are shown in the licorice representation, where S, O, C, and H atoms are yellow, red, cyan, and white, respectively.

## Conclusions

Using classical MD simulations to model the aqueous interface of a carboxylate-terminated monolayer, we investigated the binding of alkaline earth metal ions to an anionic carboxylate monolayer. A collective ion approach using the Langmuir isotherm to quantify the strength of interfacial ion adsorption is necessary to reproduce the experimental AFM results. Site-specific methods that measure the ion-carboxylate interaction for specific ion pairs qualitatively agree with experimental and computational data reported for dilute aqueous solutions of carboxylate anions such as acetate. However, they do not agree with the interfacial AFM results. Average adhesion forces overestimate the strength of Mg^2+^ ion binding relative to Ca^2+^. PMF binding free energies do not reproduce the magnitude or ion-dependent trend of AFM results. Because these methods are inherently site-specific, additional force field parametrization or enhanced sampling methods are not expected to improve these calculated results. Alternatively, the Langmuir isotherm accurately quantifies the binding free energy based on the equilibrium distribution of bound and unbound alkaline earth metal ions at the aqueous interface of a MUA monolayer. Scaling the divalent metal ion charge did not improve the quantitative agreement with the AFM results. Overall, this work demonstrates that classical force fields developed to reproduce local ion properties of alkaline earth metal ions accurately model interfacial ion adsorption when the collective ion distribution across the interface is taken into account. Future work that explicitly models the functionalized AFM tip may provide additional insight into the collective nature of ion adsorption at the aqueous interface of carboxylate-terminated monolayers.

Using the alkaline earth metal ion force field validated against AFM data, the origin of ion-specific trends in alkaline earth metal ion binding to a carboxylate-terminated monolayer was explored. Due to its strongly held first solvation shell, Mg^2+^ exhibits both direct binding (i.e., contact ion pairs) and indirect binding (i.e., solvent shared ion pairs). The larger alkaline earth metal ions of Ca^2+^, Sr^2+^, and Ba^2+^ bind primarily through direct contacts, where the portion that forms bidentate direct contacts increases with ion size. This trend is opposite that of DFT binding energies of individual ion-carboxylate complexes in aqueous solution. Analysis of the MUA monolayer reveals that the distribution of carboxylate groups within the monolayer is more varied with smaller alkaline earth metal ions. In contrast, Ba^2+^ ions effectively straddle the distance between neighboring MUA carboxylate groups, allowing for more direct contacts to be formed. These results suggest that the ion-specific trends revealed by AFM experiments may originate from the ion’s ability to efficiently occupy the carboxylate-terminated monolayer rather than the strength of individual ion-carboxylate interactions.

This work reconciles interfacial data and dilute solution data often used to interpret interfacial data by demonstrating the importance of explicitly accounting for the distribution of alkaline earth metal ions across the aqueous interface of a carboxylate terminated monolayer. Taken together, results from this work support an ion correlation mechanism modulated by ion-specific binding site interactions. These results may be utilized to tune the strength of self-assembly and adsorption of nanoscale structures facilitated by divalent metal ions, which have applications in nanotechnology. The collective distribution of alkaline earth metal ions and their efficiency at occupying the aqueous interface of fatty acid monolayers may influence the physical properties of sea spray aerosols. Additional insight into divalent metal ion adsorption may be gained by comparing atomistic simulations to concentration-dependent data from theoretical models and interfacial experiments.

## Supplementary Material



## Notes

Ion Lennard-Jones parameters; simulation convergence and validation data; experimental reference data; additional PMF free energy curves; binding motif cutoff distances; interfacial properties as a function of simulation box height; MUA carboxylate oxygen–oxygen radial distribution functions (PDF)
