# Supplementary material for: Ion-Specific Effects of Alkaline Earth Metal Ion Binding to an Anionic Carboxylate Monolayer
Source: Langmuir. 2025 Dec 24;42(1):330–43. doi: 10.1021/acs.langmuir.5c04163 (PMC12810369; doi:10.1021/acs.langmuir.5c04163)
Supplement: Supplementary file 1 [file la5c04163_si_001.pdf]

Supporting Information for

Ion-Specific Effects of Alkaline Earth Metal Ion Binding to an Anionic Carboxylate Monolayer

Lacey LaBee[†], *Kierra Parker*[†], *Audra Dempsey*[§], *Minh Tran*[§], *Gabby Delpleash*[§], *Ann Obiesie*[§], *Desirè Johnson*[†], *R. Sydney Williams*[†], *and Makenzie Provorse Long*[§]

† Department of Chemistry and Biochemistry, University of Central Arkansas, Conway, Arkansas 72035, United States

§ Department of Chemistry and Biochemistry, Creighton University, Omaha, Nebraska 68178, United States

Table of Contents

Table S1. Monovalent and divalent ion Lennard-Jones parameters. S2
Figure S1. Simulation convergence data. S3
Table S2. Number of divalent metal ions bound. S3
Table S3. Validation of solvated MUA monolayer simulation procedure. S4
Table S4. Experimental atomic force microscopy (AFM) data. S4
Figure S2. Potential of mean force (PMF) free energy curves of Mg^{2+} and Ca^{2+}. S5
Table S5. Cutoff distances (in Å) for different alkaline earth metal ion binding motifs. S5
Figure S3. Number densities as a function of simulation box height. S6
Figure S4. Interfacial properties as a function of the simulation box height. S7
Figure S5. Radial distribution function (RDF) between MUA oxygen atoms. S8
References S9

Table S1. Monovalent and divalent ion Lennard-Jones parameters.

force field	ion	σ (nm)[a]	ε (kJ/mol)
charmm36-nov2018 port for GROMACS	Na^+	0.251367073323	0.19623
	Cl^-	0.404468018036	0.62760
Li et al.[b]	Mg^{2+}	0.242300000000	0.0426867
	Ca^{2+}	0.293800000000	0.44321
	Sr^{2+}	0.322500000000	0.926018
	Ba^{2+}	0.359700000000	1.70141
Mamatkulov and Schwierz[c]	Mg^{2+}	0.238500000000	0.62000
	Ca^{2+}	0.270800000000	1.22000
Mendes de Oliveira et al.[d]	Mg^{2+}	0.136000000000	3.66100
	Ca^{2+}	0.266558000000	0.50725

a $\sigma = \mathrm{R}_{\mathrm{min}}/2^{1/6}$
b TIP3P compromise (CM) parameters from reference 1.
c Reference 2.
d Reference 3.

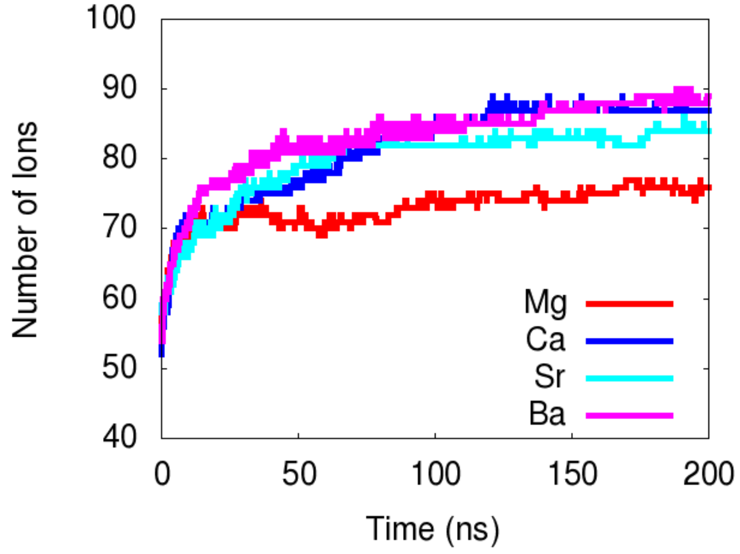

Figure S1. Number of divalent metal ions bound to MUA as a function of simulation time modeled using the Li et al. force field (see Table 1) without ECC charge scaling.

Table S2. Concentration dependent divalent metal ion adsorption.[a]

ion	100 divalent metal ions	80 divalent metal ions
Mg^{2+}	74.9 ± 0.9	66.1 ± 1.9 (82.6 ± 2.4)
Ca^{2+}	86.8 ± 0.9	78.2 ± 0.7 (97.8 ± 0.9)
Sr^{2+}	83.0 ± 0.8	75.1 ± 1.1 (93.9 ± 1.4)
Ba^{2+}	86.9 ± 1.5	75.9 ± 1.9 (94.9 ± 2.4)

a Number of divalent metal ions bound to MUA monolayer for simulations with 100 or 80 divalent metal ions. Mean and standard deviation values were calculated from the last 100 ns of each production simulation. For 80 divalent metal ions, the percentage of divalent metal ions bound is given in parentheses. Divalent metal ions within 6.0 Å of the carboxylate carbon atom are considered bound.

Table S3. Validation of solvated MUA monolayer simulation procedure.

source	Ca^{2+}-carboxylate distance (Å)[a]		MUA tilt angle (°)[b]
	bidentate	monodentate	
literature	2.9	3.5	26
this work	2.78	3.48	25

a Distance between the Ca^{2+} ion and the carboxylate carbon atom. Literature value reported from *ab initio* molecular dynamics simulations.[4]

b MUA title angle is the angle between the vector along the hydrocarbon chain of MUA and the vector normal to the monolayer surface. Literature value reported from a classical molecular dynamics simulation with monovalent Na^{+} ions only.[5] This work value reported for a classical molecular dynamics simulation with Na^{+} ions only.

Table S4. Experimental atomic force microscopy (AFM) data.

ion	F_{AD} (pN)[a]	$\lvert\Delta G_{\mathrm{bind}}\rvert$ (kJ/mol)[b]
Mg^{2+}	462 ± 9	3.12 ± 1.95
Ca^{2+}	1005 ± 11	6.78 ± 4.24
Sr^{2+}	925 ± 15	6.24 ± 3.90
Ba^{2+}	1467 ± 21	9.90 ± 6.19

a Reference 6.

b Calculated from F_{AD} data using the approach described in the Methods section of the manuscript.

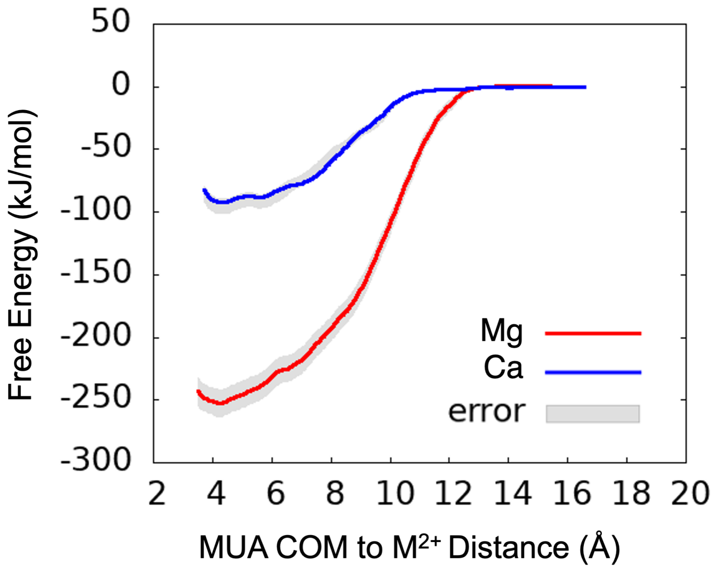

Figure S2. Potential of mean force (PMF) free energy curves of Mg^{2+} and Ca^{2+} from the center of mass (COM) of the MUA monolayer calculated using the Li et al. force field (see Table 1) with ECC charge scaling.

Table S5. Cutoff distances (in Å) for different alkaline earth metal ion binding motifs.[a]

ion	bidentate	direct	total
Mg^{2+}	2.6	3.5	6.0
Ca^{2+}	3.0	4.0	6.0
Sr^{2+}	3.2	4.3	6.0
Ba^{2+}	3.4	4.5	6.0

a Defined using carboxylate carbon-ion RDFs shown in Figure 6a.

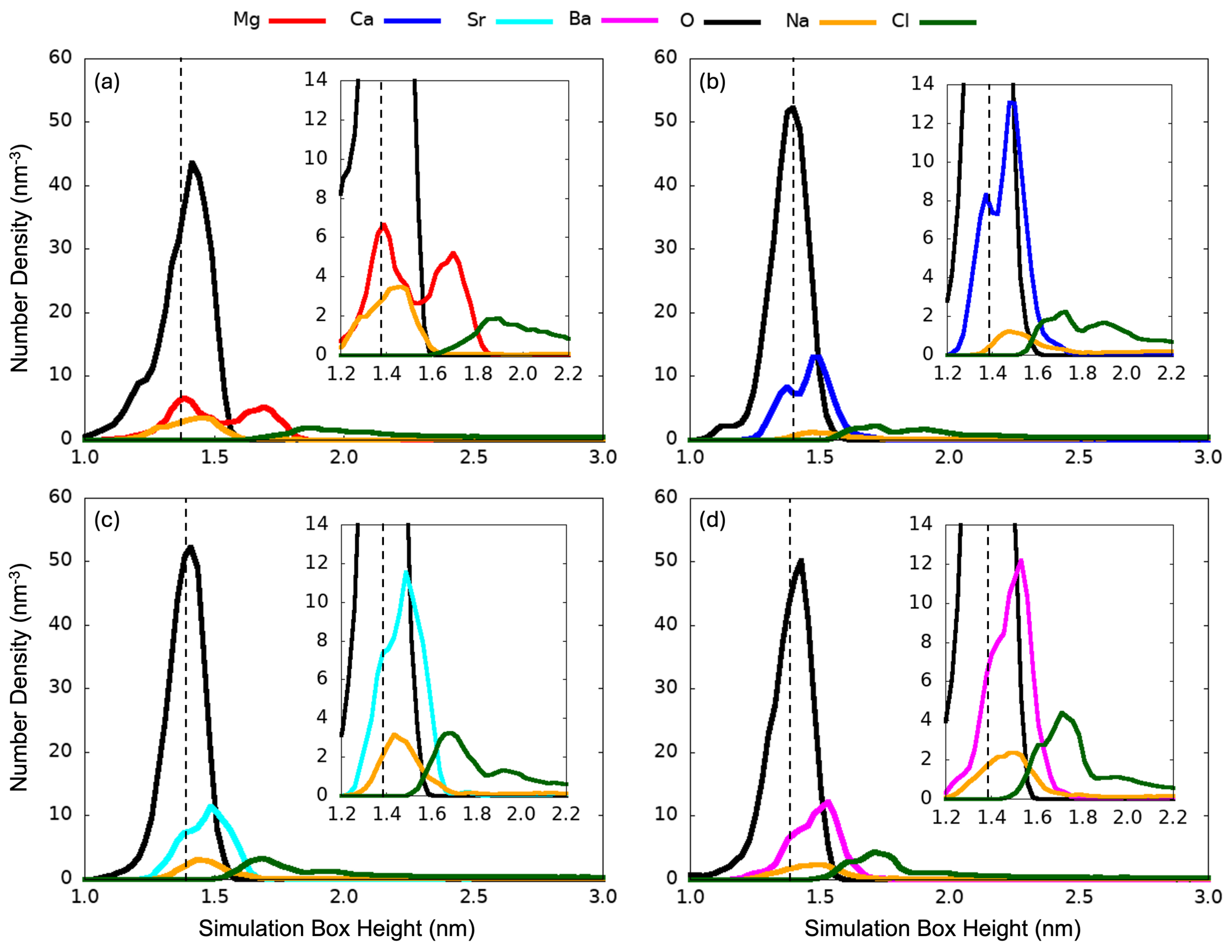

Figure S3. Number densities (nm^{-3}) as a function of the simulation box height (nm) oriented along the vector normal to the MUA monolayer surface. The mean height of carboxylate oxygen atoms is shown as a dotted line for each simulation. Insets show detailed distributions of the monovalent and divalent ions near the MUA monolayer aqueous interface.

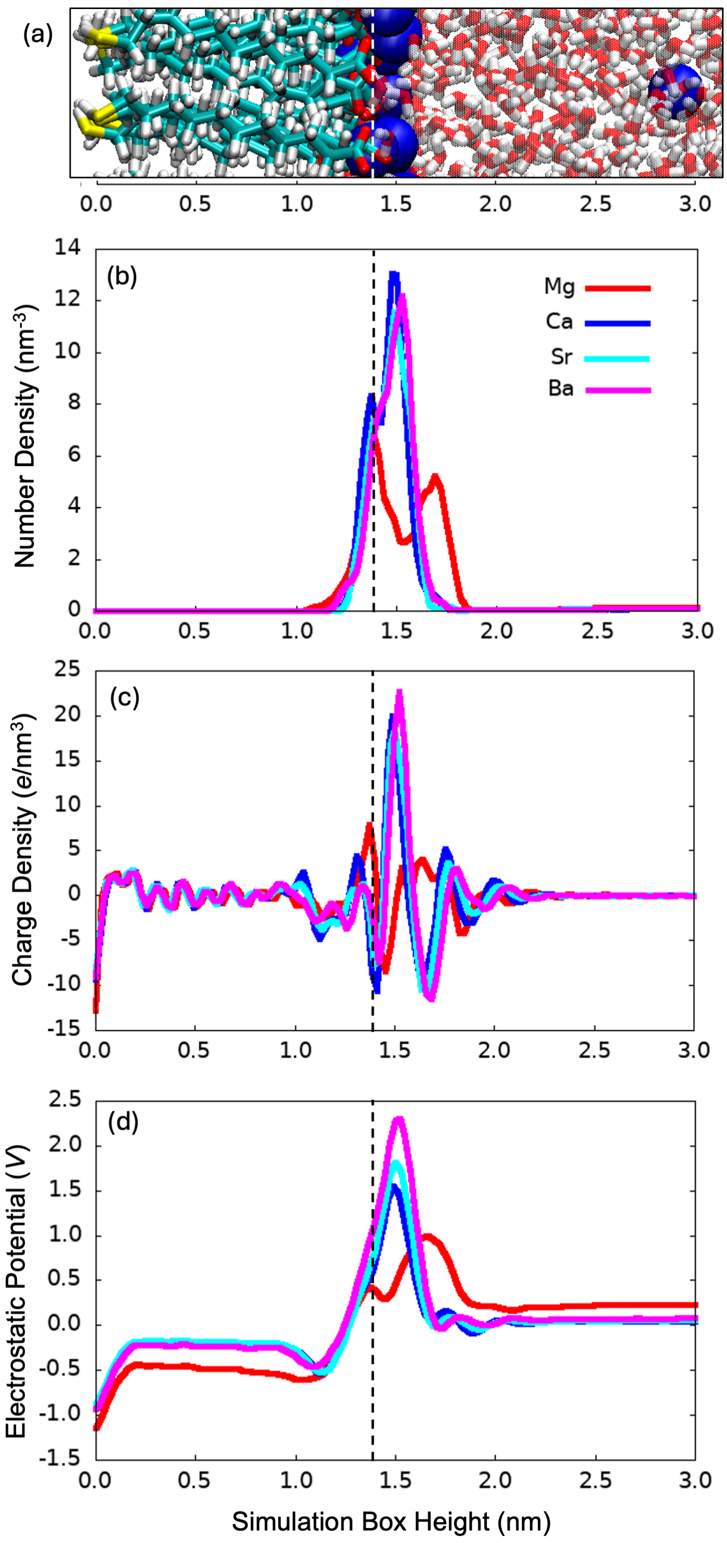

Figure S4. Interfacial properties as a function of simulation box height (nm). (a) Representative snapshot of divalent metal ion adsorption. Height of the simulation box is defined relative to the

average position of the MUA sulfur atoms. The average position of the carboxylate oxygen atoms is shown as a white dashed line. The MUA monolayer ligands are shown in the licorice representation. Ca^{2+} ions are shown as blue spheres. Water molecules are shown in the transparent licorice representation. (b) Divalent metal ion number density (nm^{-3}), (c) charge density (e/nm^3), and electrostatic potential (V) are shown as a function of the simulation box height (nm) for all four divalent metal ion simulations. The black dashed line shows the approximate average position of the carboxylate oxygen atoms for all four simulations.

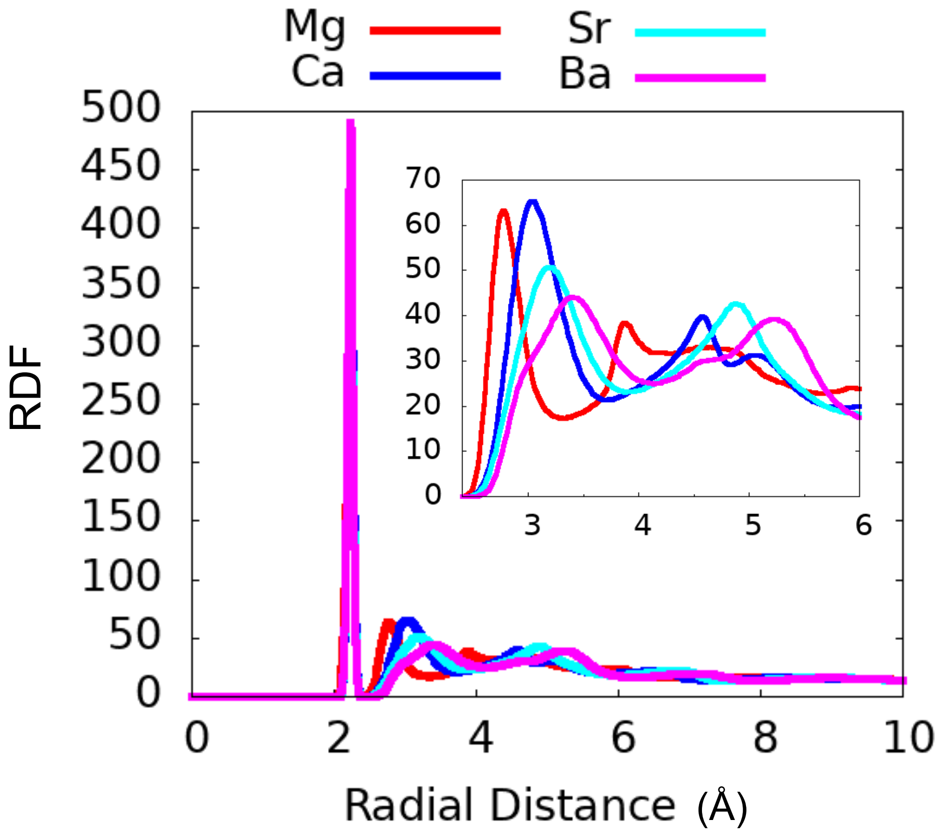

Figure S5. Radial distribution function (RDF) between oxygen atoms of the MUA carboxylate groups. Inset shows the RDF from 2.4 Å to 6.0 Å.

References

(1) Li, P.; Roberts, B. P.; Chakravorty, D. K.; Merz, K. M. Rational Design of Particle Mesh Ewald Compatible Lennard-Jones Parameters for +2 Metal Cations in Explicit Solvent. *J. Chem. Theory Comput.* **2013**, *9*, 2733–2748. https://doi.org/10.1021/ct400146w.

(2) Mamatkulov, S.; Schwierz, N. Force Fields for Monovalent and Divalent Metal Cations in TIP3P Water Based on Thermodynamic and Kinetic Properties. *J. Chem. Phys.* **2018**, *148*, 074504. https://doi.org/10.1063/1.5017694.

(3) Mendes de Oliveira, D.; Zukowski, S. R.; Palivec, V.; Hénin, J.; Martinez-Seara, H.; Ben-Amotz, D.; Jungwirth, P.; Duboué-Dijon, E. Binding of Divalent Cations to Acetate: Molecular Simulations Guided by Raman Spectroscopy. *Phys. Chem. Chem. Phys.* **2020**, *22*, 24014–24027. https://doi.org/10.1039/d0cp02987d.

(4) Martinek, T.; Duboué-Dijon, E.; Timr, Š.; Mason, P. E.; Baxová, K.; Fischer, H. E.; Schmidt, B.; Pluhařová, E.; Jungwirth, P. Calcium Ions in Aqueous Solutions: Accurate Force Field Description Aided by Ab Initio Molecular Dynamics and Neutron Scattering. *J. Chem. Phys.* **2018**, *148*, 222813. https://doi.org/10.1063/1.5006779.

(5) Szefczyk, B.; Franco, R.; Gomes, J. A. N. F.; Cordeiro, M. N. D. S. Structure of the Interface between Water and Self-Assembled Monolayers of Neutral, Anionic and Cationic Alkane Thiols. *J. Mol. Struct. THEOCHEM* **2010**, *946*, 83–87. https://doi.org/10.1016/J.THEOCHEM.2009.11.021.

(6) Rios-Carvajal, T.; Bovet, N.; Bechgaard, K.; Stipp, S. L. S.; Hassenkam, T. Effect of Divalent Cations on the Interaction of Carboxylate Self-Assembled Monolayers. *Langmuir* **2019**, *35*, 16153–16163. https://doi.org/10.1021/acs.langmuir.9b02694.
